# Delayed vs. Concomitant Urethrectomy for Non-Metastatic Urothelial Carcinoma of the Urinary Bladder Undergoing Radical Cystectomy: Perioperative and Survival Outcomes from a Single Tertiary Centre in the United Kingdom

**DOI:** 10.3390/jpm15080375

**Published:** 2025-08-14

**Authors:** Francesco Del Giudice, Mohamed Gad, Valerio Santarelli, Rajesh Nair, Yasmin Abu-Ghanem, Elsie Mensah, Ben Challacombe, Jonathan Kam, Youssef Ibrahim, Basil Lufti, Amir Khan, Akra Yeasmin, Kathryn Chatterton, Suzanne Amery, Katarina Spurna, Romerr Alao, Syed Ghazi Ali Kirmani, Felice Crocetto, Biagio Barone, Bernardo Rocco, Alessandro Sciarra, Benjamin I. Chung, Ramesh Thurairaja, Muhammad Shamim Khan

**Affiliations:** 1 Department of Maternal-Infant and Urological Sciences, “Sapienza” University of Rome, Umberto I Hospital, 00185 Rome, Italy; 2 Department of Urology, Stanford University School of Medicine, Stanford, CA 94304, USA; 3 Guy’s and St. Thomas’ NHS Foundation Trust, Guy’s Hospital, London SE1 7EH, UK; mohamed.gad@gstt.nhs.uk (M.G.); akra.yeasmin@nhs.net (A.Y.);; 4 Division of Urology, Department of Surgery, University of Maryland School of Medicine, Baltimore, MD 21201, USA; afkhan@som.umaryland.edu; 5 Department of Neurosciences, Reprodctive Sciences and Odontostomatology, University of Naples “Federico II”, 80131 Naples, Italy; 6 Department of Urology, Fondazione Policlinico Universitario A. Gemelli IRCCS, Università Cattolica del Sacro Cuore, 00136 Rome, Italy

**Keywords:** bladder cancer, radical cystectomy, urethrectomy, prophylactic urethrectomy

## Abstract

**Introduction:** The role of urethrectomy at the time of Robotic-Assisted or Open Radical Cystectomy (RARC, ORC) is controversial. Whether urethrectomy should be performed at the time of RARC/ORC or delayed up to a 3–6 month interval is unclear. We performed a retrospective cohort analysis of perioperative and survival outcomes in patients with high-risk NMIBCs or non-metastatic MIBCs at our institution who underwent either concomitant or deferred urethrectomy after RC. **Materials and Methods:** cTis-T1 or cT2-T4, N0-1, M0 BC patients who underwent RARC or ORC from 2009 to 2024 were reviewed. Clinical, demographic, tumour, and patient characteristics and perioperative variables were assessed across concomitant and delayed urethrectomy groups. Multivariate logistic analysis was performed to estimate the impact of significant variables on intraoperative and postoperative outcomes. Univariable Kaplan–Meier and multivariable Cox regression modelling was implemented to explore the relative effect of time of urethrectomy on progression-free survival (PFS), cancer-specific survival (CSS), and overall survival (OS). **Results:** A total of *n* = 58 patients (*n* = 47 delayed vs. *n* = 11 concomitant) with similar demographic characteristics were included. The concomitant urethrectomy group experienced longer operative time and greater blood loss (379 ± 65 min and 430 ± 101 mL vs. 342 ± 82 min and 422 ± 125 mL, with *p* = 0.049 and *p* = 0.028, respectively). Hospital readmission rates were higher in the concomitant urethrectomy group (36.4% vs. 8.5%, *p* = 0.016; OR: 17.9; 95% CI 1.2–265; *p* = 0.036). In Cox regression analysis, the timing of urethrectomy had no influence on PFS, CSS, or OS (all *p* > 0.05). **Conclusions:** Our study suggests that urethrectomy can be safely deferred unless urothelial disease is clearly present pre- or intraoperatively without compromising survival outcome and with the advantage of reducing surgical morbidity at the time of RC.

## 1. Introduction

Radical Cystectomy (RC) is the main treatment option for localised Muscle-Invasive Bladder Cancer (MIBC) and selected high- or very high-risk Non-Muscle-Invasive Bladder Cancer (NMIBC) [1,2]. Urothelial carcinoma is historically depicted as a pan-urothelial disease [3,4]. Therefore, the transitional epithelium of remnant ureters and urethra remains at increased risk of recurrence. Urethral recurrence (UR) after RC is a relatively rare occurrence, with a reported incidence ranging from 1% to 8% [5]. The likelihood of UR is higher in the first two postoperative years after cystectomy [6,7]. In recent years, the necessity of prophylactic or completion urethrectomy—whether performed concurrently with cystectomy or as a delayed procedure—has been increasingly debated. This reconsideration is driven by the rising adoption of orthotopic neobladder (ONB) and concerns over the added morbidity, particularly given the uncertain survival benefit associated with urethrectomy [8,9,10].

Multiple tumours, bladder neck involvement, papillary pattern, carcinoma in situ (CIS), prostatic urethral involvement, and prostatic stromal invasion are the most recognised risk factors for UR [11,12]. American Urological Association (AUA) guidelines recommend verifying a negative urethral margin before ONB and suggest avoiding ONB in patients at increased risk of UR [11]. Additionally, serial follow-up of the remnant urethra is challenging, and standardised risk-adjusted surveillance is lacking [13]. Moreover, should UR be diagnosed, there is a lack of evidence-based recommendations regarding its optimal management [5,14]. Therefore, in case of multiple risk factors and a non-orthotopic urinary diversion, a completion urethrectomy remains a valid treatment choice, while mandatory in the case of a positive frozen urethral section or obvious synchronous tumours in the urethra diagnosed before cystectomy.

Prophylactic urethrectomy can be performed either at the time of RC (immediate or concomitant urethrectomy) or can be deferred to a later date, generally 1–4 months after RC (delayed or staged urethrectomy) [15]. The decision to perform a prophylactic delayed urethrectomy can be taken after the availability of the final pathological report, in view of the confirmed risk factors for UR, such as a positive urethral margin or a CIS in the prostatic urethra [16]. The immediate urethrectomy can be performed en bloc with the cystoprostatectomy by a prepubic approach or via a separate perineal incision, whereas a delayed urethrectomy is generally performed through a perineal approach. Currently, clear guidance on indications and timing of prophylactic urethrectomy is lacking. While suggesting the consideration for a radical urethrectomy in the case of positive urethral margins or clear urethral involvement, whether urethrectomy should be performed at the time of cystectomy or delayed is not mentioned in the current European Association of Urology (EAU) guidelines [17].

Few studies have compared surgical morbidity and long-term survival of concomitant and delayed urethrectomy. In theory, the total duration of hospitalisation should be shorter when a concomitant approach is chosen. However, it adds approximately an additional hour to an already long and demanding operation if performed by a single surgeon or else has to involve an extra surgeon to perform urethrectomy simultaneously. There are added technical difficulties due to restricted access while the patient is placed in the lithotomy position for the cystectomy part of the operation. There is also an increased risk of postoperative complications including deep venous thrombosis [18,19].

On the other hand, a delayed urethrectomy could be technically more difficult, especially at the level of the urethral stump, due to postoperative fibrotic changes [18,20]. However, currently available data is scarce and mostly based on limited case series. In the present study, we aimed to compare surgical morbidity and long-term survival outcomes of concomitant versus delayed completion urethrectomy.

## 2. Materials and Methods

### 2.1. Study Cohort Characteristics According to Urethrectomy Timing

Patients with a histologically confirmed diagnosis of non-metastatic (i.e., cN0, M0) localised MIBC or high-risk/very high-risk NMIBC who underwent RC from 2009 to 2024, with prophylactic urethrectomy, either concomitantly or delayed, were retrospectively reviewed. The clinical indications for urethrectomy included a positive urethral margin, multifocal tumours, CIS into prostatic urethra, and prostatic ductal or stromal involvement. Demographic, anthropometric, and clinical characteristics (age, Body Mass Index [BMI], gender, Charlson Comorbidity Index [CCI], American Society of Anaesthesiologists [ASA] score) were recorded. A formal Multidisciplinary Team (MDT) discussion prior to being offered RC ± prophylactic urethrectomy as primary treatment was always performed. In all cases of delayed urethrectomy, the final pathological report was re-discussed in the MDT to confirm the indication for prophylactic urethrectomy. After appropriate counselling regarding possible management options (surveillance vs. urethrectomy), all included patients signed the informed consent for the procedure.

Inclusion criteria were the absence of distant metastases and lymph-node involvement at clinical or pathological staging, a histologically confirmed diagnosis of urothelial or mixed variant BC obtained by at least one previous TURBT, and RC plus concomitant or delayed urethrectomy with or without Neoadjuvant Chemotherapy (NAC) performed with curative intent. Previous External Beam Radiation Therapy (EBRT), chemotherapy, life expectancy < 5 years, and RC cases performed with other indications rather than curative intent for localised cN0M0 BC were excluded. Also, patients undergoing urethrectomy for pathologically proven urethral recurrence were excluded. Preoperative clinical stage was assigned by a single or combination of diagnostic tool assessments, including cystoscopy, TURBT, Computerised Tomography (CT), multiparametric Magnetic Resonance Imaging (mpMRI), and Fluodo-Deoxyglucose Positron Emission Tomography (FDG-PET).

### 2.2. Surgical Procedure and Pathological Analysis

All procedures were performed by the same team of three experienced consultants (MSK, RT, RN) in the setting of a UK-certified Senior Robotic Clinical Fellowship program. RC performed via both an open (Open Radical Cystectomy [ORC]) or Robotic (Robotic-Assisted Radical Cystectomy [RARC]) approach was included. Either intracorporeal or extracorporeal Ileal Conduit (IC) or Ureterocutaneostomy (UCS) was performed as urinary diversions (UD). Pelvic Lymphadenectomy (PLND) was carried out with a standard or extended template following appropriate MDT assessment. Frozen section analysis of the urethra and ureters during RC was performed in those cases initially scheduled for an ONB. The decision to perform immediate or delayed urethrectomy was at the discretion of the attending surgeon. In addition, intraoperative factors not strictly related to tumour characteristics, such as intraoperative distress caused by prolonged anaesthesia or Trendelenburg position, could have influenced the operating team’s decision to postpone the urethrectomy. A simultaneous urethrectomy was performed by a perineal or a prepubic approach, while all the delayed urethrectomies were carried out via a perineal incision.

Final histology and pathological stage were reported according to the American Joint Committee on Cancer (AJCC) Guidelines. All the RC and urethrectomy specimens were analysed by experienced uropathologists at our institution with >20 years of experience in Urothelial Cancers. pT and pN stage, tumour grade, histological variants, and concomitant CIS and lymph-vascular invasion (LVI) were included in the final pathological report.

All patients were followed at regular intervals with clinical and radiological follow-up to determine time to progression and survival outcomes. In case of a local or distant recurrence, it was managed according to specific guidelines and MDT assessment.

### 2.3. Statistical Analysis

Statistical analysis was carried out according to previously described methodology [21]. Descriptive statistics were used to summarize pertinent study information. Categorical data was reported as numbers and percentages, and continuous variables were expressed as median, Interquartile Range (IQR), mean, and standard deviation (SD). The Pearson Chi-square test or Fisher’s exact test was used to test association between variables. A Mann–Whitney test or ANOVA one-way test was performed when analysing quantitative data and pairwise intergroup comparisons of variables. Subsequently, a set of multivariable regression models was developed to estimate the impact of relevant perioperative variables on the risk of intraoperative complications, postoperative complications, and readmissions. Kaplan–Meier survival analyses and log-rank test were used to explore the univariate effect of the time of urethrectomy on CSS and OS. Subsequently, multivariable Cox regression models were adopted to estimate Hazard Ratios (HR) of selected relevant variables for progression-free survival (PFS), cancer-specific survival (CSS), and overall survival (OS). Statistical analysis was carried out using SPSS Statistics (IBM Corp: Armonk, NY, USA) version 27.0 and Stata version 18.1 (Stata Corporation, College Station, TX, USA) with statistical significance set as *p* < 0.05.

## 3. Results

### 3.1. Study Cohort Characteristics According to Urethrectomy Time

Baseline characteristics of the cohort, sub-divided according to the timing of urethrectomy (concomitant vs. delayed), are shown in Table 1. A total of *n* = 58 patients were included in the study. Of these, *n* = 47 (*n* = 45 males and *n* = 2 females) were in the delayed urethrectomy cohort, and *n* = 11 (*n* = 7 males and *n* = 4 females) were in the concomitant urethrectomy group.

TURBT histology and perioperative endoscopic findings were explored to investigate factors influencing the choice of a concomitant vs. delayed urethrectomy approach. A High Grade (HG) tumour from the TURBT specimen found similar representation in most of the whole cohort (*n* = 45, 95.7%, and *n* = 11, 100%, from delayed and concomitant urethrectomy, respectively, *p* = 0.486). RC was performed in patients with previous BC history in *n* = 18 (38.3%) cases in the delayed and *n* = 2 (18%) cases in the concomitant urethrectomy group (*p* = 0.206), while no differences in previous BCG exposure were detected.

Preoperative clinical stage was, however, significantly higher in patients treated with a delayed urethrectomy approach. In particular, those undergoing concomitant urethrectomy were more frequently affected by high-risk NMIBC (*n* = 6 pTa, 54.5%, *n* = 1 pT1, 9.1%, and *n* = 4 pTis, 36.4%) than those in the delayed urethrectomy group (*n* = 7 pTa, 14.9%, *n* = 21 pT1, 44.7%, and *n* = 4 pTis, 8.5%). On the other hand, 31.9% of patients in the delayed urethrectomy group had a T2 tumour at the previous TURBT when compared to concomitant urethrectomy patients who were never clinically classified as MIBC (*p* < 0.0001).

Over the study period, robotic RC (RARC) was the approach of choice in most cases, regardless of the decision to perform a concomitant or delayed urethrectomy (83% and 90.9%, respectively). This was also true for intracorporeal UD, which was performed in *n* = 36 (76.6%) and *n* = 9 (81.8%) patients in the delayed and concomitant urethrectomy cohorts, respectively.

As expected, patients who underwent a concomitant urethrectomy had significantly longer operative times and higher blood loss (342 ± 82 min and 422 ± 125 mL in delayed urethrectomy vs. 379 ± 65 min and 430 ± 101 mL in concomitant urethrectomy, *p* = 0.049 and *p* = 0.028).

Positive intraoperative frozen sections of urethra were found in *n* = 3 (6.4%) of cases in the delayed urethrectomy cohort and *n* = 2 (18.2%) in the concomitant urethrectomy group (*p* = 0.209). Of note, while these patients were originally scheduled to receive an ONB, the diversion of choice was adapted to IC or UCS and a urethrectomy following the positive frozen section notification.

No significant differences in terms of intraoperative and postoperative complications were found between the two groups (*p* = 0.639 and *p* = 0.09). Most postoperative complications were Clavien Dindo (CD) grade 1 to 3, with only one CD 4 and no CD 5 complications. Mean cumulative hospitalisation stay was longer in the delayed urethrectomy cohort, but the difference did not reach significance (8.7 ± 4.6 days vs. 6.6 ± 4.21 days, *p* = 0.11). Readmission rate was significantly higher in the concomitant urethrectomy group than in the delayed urethrectomy group (36.4% vs. 8.5%, respectively, *p* = 0.016).

Interestingly, no significant differences in pathological findings at the RC specimen (*p* = 0.307) were detected. *n* = 10 patients (90.9%) in the concomitant urethrectomy group were found to have either a pTis (*n* = 6, 54.5%) or a pT1 (*n* = 4, 36.4%) tumour. In the delayed urethrectomy group, *n* = 2 (4.3%), *n* = 2 (4.3%), *n* = 15 (31.9%), and *n* = 9 (19.1%) patients had a pathological stage at the final RC specimen of pT0, pTa, pTis, and pT1, respectively. A MIBC at final pathology was found in *n* = 1 (9.1%) patient in the concomitant urethrectomy group and *n* = 19 (40.4%) patients in the delayed urethrectomy group (*n* = 15, 31.9% pT2, and *n* = 4, 8.5% pT3). A CIS in the urethral or ureteral stump of the bladder specimen was found in *n* = 29 (61.7%) and *n* = 13 (27.7%) patients in the delayed urethrectomy group and in *n* = 2 (18.2%) and *n* = 0 patients in the concomitant urethrectomy group (*p* = 0.009 and *p* = 0.048).

The rates of positive urethral pathological results were similar between the two groups (32% vs. 27.3% for the delayed and concomitant urethrectomy groups, respectively, *p* = 0.4).

Recurrence rates were similar in the two groups (*n* = 17, 36.2%, recurrences in delayed urethrectomy and *n* = 2, 18.2%, recurrences in the concomitant urethrectomy) with a mean time to recurrence of 32.7 ± 25.8 months and 81.5 ± 9.2 months in the delayed urethrectomy and concomitant urethrectomy groups, respectively (*p* = 0.063).

### 3.2. Multivariable Logistic Modelling for Perioperative Outcomes

Results of multivariate regression exploring the impact of preoperative variables on perioperative and postoperative outcomes are shown in Table 2. The only variable significantly and independently associated with an increased risk of intraoperative complications was CCI (OR: 1.8; 95% CI 1.1–3; *p* = 0.025). The variable of interest (i.e., concomitant vs. delayed urethrectomy) did not significantly influence the risk of intraoperative complications (OR: 3; 95% CI 0.3–32; *p* = 0.3). No single confounder significantly influenced the risk of postoperative complications (*p* > 0.05). A concomitant urethrectomy was found to be the only independent risk factor for readmissions (OR: 17.9; 95% CI 1.2–265; *p* = 0.036).

### 3.3. Survival Analysis According to Urethrectomy Time

#### 3.3.1. Kaplan–Meier Analysis for CSS

**Figure 1:**
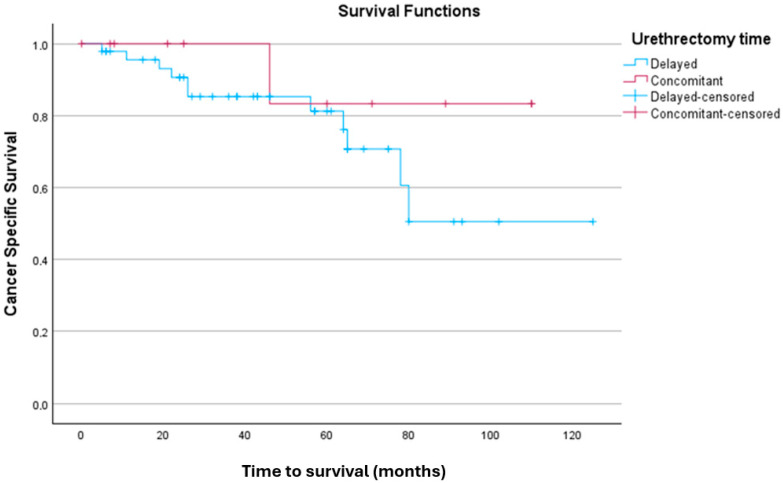
Kaplan–Meier analysis comparing CSS distributions between the delayed urethrectomy and concomitant urethrectomy groups.

A Kaplan–Meier survival analysis was applied to explore the univariate effect of urethrectomy timing on CSS distributions (Figure 1). The case processing summary indicated that out of 47 patients in the delayed urethrectomy group, 11 experienced the event of interest, while 36 were censored (76.6%). In the concomitant urethrectomy group, 1 out of 11 patients experienced the event, with 10 censored cases (90.9%). Overall, 79.3% of the data was censored, indicating patients for whom the event did not occur during the range of study time. CSS times were estimated for both groups. For the delayed urethrectomy group, the mean survival time was 90.93 months (SE = 8.23, 95% CI [74.80, 107.06]). In the concomitant urethrectomy group, the mean survival time was 99.33 months (SE = 9.74, 95% CI [80.25, 118.42]). Overall, the mean CSS time across all participants was 95.38 months (SE = 7.08, 95% CI [81.50, 109.26]).

The log-rank test was used to examine the equality of CSS distributions between the urethrectomy timing groups. The results indicated no significant difference between the groups, χ^2^(1) = 1.064, *p* = 0.302. Additional tests, including the Breslow (Generalised Wilcoxon) test (χ^2^(1) = 0.770, *p* = 0.380) and the Tarone–Ware test (χ^2^(1) = 0.879, *p* = 0.348), similarly showed no significant differences in CSS between the two groups.

#### 3.3.2. Kaplan–Meier Analysis for OS

**Figure 2:**
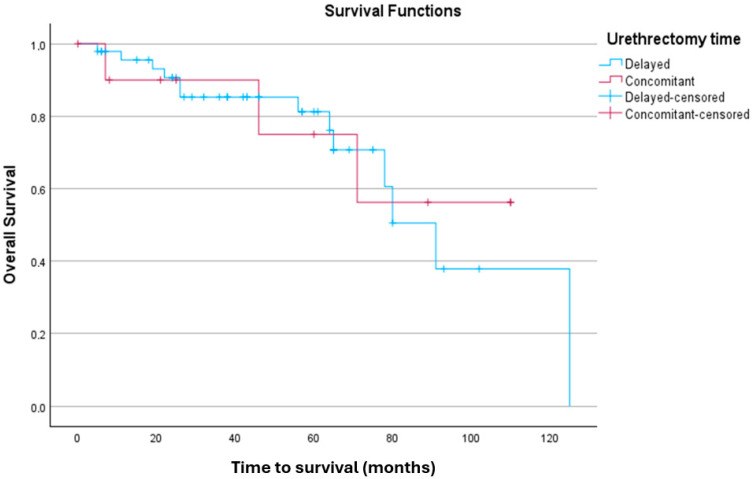
Kaplan–Meier analysis comparing OS distributions between the delayed urethrectomy and concomitant urethrectomy groups.

Kaplan–Meier survival analysis to compare OS distributions between patients undergoing delayed urethrectomy and those undergoing concomitant urethrectomy is shown in Figure 2. The case processing summary indicated that out of 47 participants in the delayed urethrectomy group, 13 experienced the event of interest, while 34 were censored (72.3%). In the concomitant urethrectomy group, 3 out of 11 participants experienced the event, with 8 censored cases (72.7%). Overall, 72.4% of the data was censored, indicating patients for whom the event did not occur during the study. OS times were estimated for both groups. For the delayed urethrectomy group, the mean survival time was 86.64 months (SE = 8.41, 95% CI [70.16, 103.12]). In the concomitant urethrectomy group, the mean survival time was 82.79 months (SE = 12.61, 95% CI [58.07, 107.51]). Overall, the mean survival time across all participants was 88.07 months (SE = 7.38, 95% CI [73.60, 102.54]).

The equality of OS distributions between the urethrectomy timing groups was explored using the log-rank test. The results showed no significant difference between the groups, χ^2^(1) = 0.030, *p* = 0.863. Additional tests, including the Breslow (Generalised Wilcoxon) test (χ^2^(1) = 0.024, *p* = 0.877) and the Tarone–Ware test (χ^2^(1) = 0.002, *p* = 0.967), similarly indicated no significant differences in OS between the two groups.

#### 3.3.3. Multivariable Cox Regression Models for PFS, CSS, and OS

Table 3 shows multivariable Cox regression models for the prediction of PFS, CSS, and OS. None of the explored variables, including urethrectomy time (delayed vs. concomitant), positive frozen section of urethra, urethral CIS, LVI, and pT stage at RC, were found to be significant and to independently predict PFS, CSS, and OS (*p* > 0.05).

## 4. Discussion

Prophylactic urethrectomy is an infrequently performed procedure, due to the growing diffusion of readily available follow-up tools, such as flexible urethrocystoscopy, urethral wash cytology, and pelvic mpMRI in cases with suspected urethral recurrence [22,23,24,25]. In addition, the robotic approach allows for a more extended membranous urethral dissection with a secure closure of the urethra to prevent spillage [26]. Concomitant prophylactic urethrectomy has a potential increased risk of morbidity, including wound infection, scrotal hematoma, scrotal oedema, and DVT due to lithotomy position [27,28]. Urethral preservation, however, may be associated with increased risk of UR, local dissemination, and recurrence, caused possibly by spillage of contaminated urine into the pelvis and/or intraperitoneal space, due to inadequate urethral ligation or undetected cancer at the urethral transection site [22].

Patients with an increased risk of UR or those with a positive urethral margin would benefit from a completion urethrectomy, as urethral recurrences can present late at an incurable stage with fatal outcomes. The decision to perform a concomitant or a delayed urethrectomy is usually based on the surgeon’s preference, due to the lack of clear guidelines and advantages of one option over the other. In the present study, we sought to compare preoperative clinical and pathological characteristics, as well as intraoperative and postoperative outcomes, of delayed vs. concomitant urethrectomy.

In our study, we did not find a clear distinction in preoperative clinical features between the two groups. The main differences were found in gender and preoperative staging. Regarding gender, a recent meta-analysis demonstrated a significantly higher risk of UR in male patients compared to their female counterparts [6]. Hence, male patients who are at increased risk of UR are more likely to be offered a prophylactic urethrectomy. This was also the case in our series, with only 10% female patients, which is a significantly lower rate than that usually reported in RC cohorts, as per a recent meta-analysis [29]. Moreover, the higher size of the delayed prophylactic urethrectomy cohort, a particularly rare choice for female BC patients, contributed to further lowering the F to M ratio. Indeed, the higher proportion of female patients in the concomitant urethrectomy group in our series is explained by the relative ease of urethrectomy, due to the shorter length of the female urethra. This is particularly true with the robotic approach, with minimal impact on the duration of the procedure and surgical morbidity. Only 11 patients underwent a concomitant urethrectomy in our cohort, and none of them were diagnosed with MIBC at the time of Radical Cystectomy but rather had refractory high-risk and very high-risk NMIBC. These patients had a high rate of CIS at TURBT (63.6%) and likely multifocal tumours, which led to a higher risk of urethral involvement even in the case of a negative urethral resection margin. Moreover, non-tumour-related intraoperative factors, such as respiratory or circulatory issues caused by the Trendelenburg positioning, could have influenced the choice of the OR team to postpone the urethrectomy to a later stage.

While analysing intraoperative outcomes, we found that a concomitant urethrectomy significantly increased operative time and estimated blood loss. Increased operating times, particularly for elderly patients and in a Trendelenburg position, are associated with increased postoperative discomfort and higher complication rates [30]. A Trendelenburg position also provides poorer urethral access and exposure. Hence, reducing total operative time by arranging the prophylactic urethrectomy for a later time would be less demanding for both patients and surgeons. Despite the relatively small sample size and lack of differences in intraoperative and postoperative complications, there was a significantly higher readmission rate in patients undergoing a concomitant urethrectomy. This could be associated with the added morbidity, pain, and reduced mobility after urethrectomy, which adds to the already challenging convalescence of RC. However, a plausible advantage of concomitant urethrectomy could be reduced cumulative length of stay, which is significantly shorter in the concomitant urethrectomy group. Additionally, this approach could be particularly convenient for those hard-to-reach patients with poor follow-up compliance.

Regarding final pathological analysis, patients in the delayed urethrectomy group in our study were found to have significantly higher rates of CIS in the urethral and ureteral stumps of the bladder specimen. These findings led the MDT to propose a prophylactic urethrectomy even for cases in which it had not been considered before RC. Nonetheless, the rates of patients having no evidence of any tumour (pT0) on the final urethral pathologic examination were comparable in the two groups and in line with those reported in previous studies [6].

Finally, we compared survival outcomes of patients in the concomitant and delayed urethrectomy groups and found no significant differences in terms of PFS, CSS, and OS. Our results are in line with those of a previous retrospective study of 76 RCs [17]. With the appropriate caution required due to the retrospective nature and small sample sizes of the studies, they suggest that a prophylactic urethrectomy can be safely delayed up to 2–6 months after the RC without negatively impacting survival outcomes.

Our study has several limitations. It is a retrospective analysis on a cohort of patients of RC who underwent prophylactic urethrectomy, but the choice to undergo a concomitant or delayed urethrectomy was mainly based on the surgeons’ preference and recommendation from the MDT. This resulted in significant clinical differences between the two groups, which could have had an unpredictable effect on the outcomes of interest. Furthermore, the relatively small sample size, especially for the concomitant urethrectomy group, could have concealed significant differences that were not highlighted in the present study.

## 5. Conclusions

Completion urethrectomy is a rarely performed procedure, with limited but well-defined indications. The rationale behind the choice to stage a prophylactic urethrectomy to a later date is to reduce operative times, as well as morbidity, and the postoperative pain associated with RC, at the cost of a second hospitalisation and additional anaesthesia. The results of our retrospective study on 58 patients undergoing Radical Cystectomy with prophylactic urethrectomy suggest that while delayed urethrectomy may increase the cumulative length of stay, it might reduce perioperative morbidity without compromising survival outcomes. However, despite ours being one of the largest available studies comparing concomitant versus delayed urethrectomy, the small sample size, particularly for the concomitant urethrectomy group, and the retrospective nature of our research impose caution in the overinterpretation of our findings. Larger-size and preferably prospective studies are required before drawing definite conclusions.

## Figures and Tables

**Table 1:** Demographic, perioperative, and pathological characteristics of the study cohort reviewed according to urethrectomy time (delayed vs. concomitant).

Urethrectomy Time	Delayed	Concomitant	*p* Value
Total	47	11	
Gender, *n* (%)			
Male	45 (95.7)	7 (63.6)	0.002
Female	2 (4.2)	4 (36.4)	0.002
Smoking habit, *n* (%)			
None	15 (31.9)	5 (45.5)	0.495
Ex smoker	22 (46.8)	3 (27.3)	0.495
Current smoker	10 (21.3)	3 (27.3)	0.495
Family history, *n* (%)			
No	47 (100)	10 (90.9)	0.037
Yes	0 (0)	1 (9.1)	0.037
Age, mean ± SD	71.91 ± 7.35	61.18 ± 12.28	0.08
BMI, mean ± SD	26.52 ± 3.66	24.96 ± 2.37	0.117
CCI, mean ± SD	4.62 ± 1.84	5.27 ± 1.80	0.149
Recurrent bladder cancer, *n* (%)	18 (38.3)	2 (18.2)	0.206
Previous BCG therapy, *n* (%)	14 (29.8)	2 (18.2)	0.438
Previous MMC treatment, *n* (%)	8 (17)	1 (9.1)	0.513
TURBT grade 1973, *n* (%)			
G2	2 (4.3)	1 (9.1)	0.514
G3	45 (95.7)	10 (90.9)	0.514
TURBT grade 2004, *n* (%)			
LG	2 (4.3)	0 (0)	0.486
HG	45 (95.7)	11 (100)	0.486
CIS at TURBT, *n* (%)			
No	16 (34)	4 (36.4%)	0.71
Yes	31 (66)	7 (63.6%)	0.71
T stage at TURBT, *n* (%)			
pTa	7 (14.9)	6 (54.5)	<0.0001
pTis	4 (8.5)	4 (36.4)	<0.0001
pT1	21 (44.7)	1 (9.1)	<0.0001
pT2	15 (31.9)	0 (0)	<0.0001
RC approach, *n* (%)			
Open	8 (17)	1 (9.1)	0.513
Robotic	39 (83)	10 (90.9)	0.513
Intracorporeal UD, *n* (%)	36 (76.6)	9 (81.8)	0.708
Extended PLND template, *n* (%)	19 (40.4)	1 (9.1)	0.049
Total LNs removed, mean ± SD	13.68 ± 6.57	12.36 ± 1.80	0.543
Blood loss (ml), mean ± SD	422.13 ± 125.74	430.00 ± 101.48	0.028
Operative time (min), mean ± SD	342.7 ± 82.7	379.6 ± 65.7	0.049
Positive frozen section on ureters, *n* (%)	5 (10.6)	0 (0)	0.258
Positive frozen section on urethra, *n* (%)	3 (6.4)	2 (18.2)	0.209
Intraoperative complications, *n* (%)	6 (12.8)	2 (18.2)	0.639
Postoperative complications, *n* (%)	16 (34)	1 (9.1)	0.09
Postoperative complications, CD, *n* (%)			
I	5 (10.6)	0	
II	8 (17)	0	0.3
III	2 (4.3%)	1 (9.1%)	
IV	1 (2.1%)	0	
V	0	0	
Cumulative length of stay (d), mean ± SD	8.7 ± 4.62	6.6 ± 4.21	0.11
Readmissions, *n* (%)	4 (8.5)	4 (36.4)	0.016
Urethrectomy months, mean ± SD	3.12 ± 2.23	NA	NA
Stage at RC, *n* (%)			
pT0	2 (4.3)	0 (0)	0.307
pTa	2 (4.3)	0 (0)	0.307
pTis	15 (31.9)	6 (54.5)	0.307
pT1	9 (19.1)	4 (36.4)	0.307
pT2	15 (31.9)	1 (9.1)	0.307
pT3	4 (8.5)	0 (0)	0.307
Concomitant CIS, *n* (%)	39 (17)	6 (45.5)	0.042
CIS on Ureters, *n* (%)	13 (27.7)	0 (0)	0.048
LVI, *n* (%)	7 (14.9)	1 (9.1)	0.615
CIS on Urethra, *n* (%)	29 (61.7)	2 (18.2)	0.009
pT0 at final urethral specimen, *n* (%)	32 (68)	8 (72.7)	0.4
Recurrence, *n* (%)	17 (36.2)	2 (18.2)	0.252
Time to recurrence (months), mean ± SD	32.72 ± 25.76	81.5 ± 9.19	0.063

BMI: Body Mass Index; SD: standard deviation; CCI: Charlson Comorbidity Index; BCG: Bacillus Calmette–Guerin; MMC: Mitomycin C; TURBT: Transurethral Resection of Bladder Tumour; LG: Low Grade; HG: High Grade; CIS: carcinoma in situ; RC: Radical Cystectomy; UD: urinary diversion; PLND: Pelvic Lymphadenectomy; LN: lymph node; LVI: lymph-vascular invasion; CD: Clavien Dindo classification.

**Table 2:** Multivariate regression models showing the risk of intraoperative complications, perioperative complications, and readmissions based on multiple predictors.

	Intraoperative Complications			Postoperative Complications			Readmissions		
Parameter	OR	95% CI	*p* Value	OR	95% CI	*p* Value	OR	95% CI	*p* Value
Urethrectomy time (concomitant)	3	0.3–32	0.3	0.16	0.01–1.6	0.13	17.9	1.2–265	0.036
BMI	1.22	0.9–2.17	0.2	0.96	0.8–1.1	0.7	0.73	0.5–1.04	0.08
CCI	1.8	1.1–3	0.025	1.1	0.8–1.5	0.6	0.74	0.4–1.4	0.3
Gender	0.13	0.03–5.15	0.3	0.4	0.02–4	0.4	1.03	0.06–17	0.9
Smoking habit (former or active)	1	0.1–10	0.9	1.05	0.2–4	0.9	4	0.5–34	0.2
RC approach (robotic)	3	0.1–100	0.5	0.3	0.04–1.7	0.17	0.5	0.01–31	0.7
EBL	1.003	1.0–1.007	0.03	1	1–1	0.9	0.99	0.98–1	0.1
Operative Time	1	0.99–1.01	0.5	1.002	0.99–1.01	0.4	0.99	0.98–1.01	0.6

OR: Odds Ratio; 95% CI: 95% Confidence Interval; BMI: Body Mass Index; CCI: Charlson Comorbidity Index; EBL: estimated blood loss.

**Table 3:** Multivariable Cox regression models for the prediction of progression-free survival, cancer-specific survival, and overall survival.

	Progression-Free Survival			Cancer-Specific Survival			Overall Survival		
	HR	95% CI	*p* Value	HR	95% CI	*p* Value	HR	95% CI	*p* Value
Urethrectomy time	4.4	0.45–42.37	0.199	2.95	0.26–33.53	0.382	0.827	0.16–4.075	0.816
Positive frozen section on urethra	2.2	0.27–17.85	0.461	0.63	0.07–5.39	0.672	1.83	0.28–11.73	0.52
Urethral CIS	1.5	0.26–9.73	0.642	2.15	0.249–22.52	0.551	1.68	0.16–14.45	0.632
LVI	-*	-*	-*	-*	-*	-*	0.72	0.07–7.33	0.782
pT Stage at RC									
pTa	Ref								
pTis	1.52	0.19–11.8	0.691	-*	-*	-*	-*	-*	-*
pT1	3.84	0.43–34.9	0.228	-*	-*	-*	-*	-*	-*
pT2	0.75	0.08–6.66	0.799	-*	-*	-*	-*	-*	-*
pT3	1.11	0.1–12.37	0.930	-*	-*	-*	-*	-*	-*

HR: Hazard Ratio; 95% CI: 95% Confidence Interval; LVI: lymph-vascular invasion; RC: Radical Cystectomy. * Too few data for reasonable computing.

## Data Availability

The data presented in this study are available on request from the corresponding author.
